# Molecular diversity of antimicrobial effectors in the oyster *Crassostrea gigas*

**DOI:** 10.1186/1471-2148-10-23

**Published:** 2010-01-25

**Authors:** Paulina Schmitt, Yannick Gueguen, Erick Desmarais, Evelyne Bachère, Julien de Lorgeril

**Affiliations:** 1 Ifremer, CNRS, Université de Montpellier II, IRD, UMR 5119 « Ecosystèmes Lagunaires », Place Eugène Bataillon, CC80, 34095 Montpellier Cedex 5 (France; 2 Institut des Sciences de l'Evolution, Université de Montpellier II, CNRS, UMR 5554. Place Eugène Bataillon, CC63, 34095 Montpellier Cedex 5 (France; 3 Ifremer, Centre Océanologique du Pacifique. BP 7004-Taravao, 98719, Tahiti-French Polynesia

## Abstract

**Background:**

To gain insight into the molecular diversity of antimicrobial peptides and proteins in the oyster *Crassostrea gigas*, we characterized and compared the sequence polymorphism of the antimicrobial peptides (AMPs), *Cg*-Defensins (*Cg*-Defs) and *Cg*-Proline Rich peptide (*Cg*-Prp), and of the bactericidal permeability increasing protein, *Cg*-BPI. For that, we analyzed genomic and transcript sequences obtained by specific PCR amplification and *in silico *searches.

**Results:**

High diversification among the three antimicrobial effectors was evidenced by this polymorphism survey. On the basis of sequence phylogenies, each AMP aggregates into clearly defined groups of variants and is the product of a multigenic family displaying a variety of gene structures. In contrast, Cg-*bpi *forms a single group and is encoded by a single gene copy. Moreover, we identified for both AMPs several genetic mechanisms of diversification such as recombination, parallel mutations leading to phylogenetic homoplasy and indel events. In addition, the non synonymous to synonymous substitutions ratio by codon (dN/dS) revealed several negatively and positively selected sites for both AMPs, suggesting that directional selection pressures have shaped their sequence variations.

**Conclusions:**

This study shows for the first time in a mollusc that antimicrobial peptides and proteins have been subject to distinct patterns of diversification and we evidence the existence of different evolutionary routes leading to such sequence variability.

## Background

The Pacific oyster, *Crassostrea gigas*, is a filter feeder bivalve mollusc which is continually exposed to microorganisms naturally present in the marine environment. Thus, the capacity to control the microflora and overcome infections is essential for the oyster survival. The hemocytes, immunocompetent cells, play a central role in innate antimicrobial response. Indeed, in molluscs, hemocytes infiltrate injured tissues, phagocyte microorganisms, produce antimicrobial peptides (AMPs) and release factors such as lectins and reactive oxygen species (ROS) [1-3].

In *C. gigas*, several antimicrobial effectors have been recently characterized. An AMP, member of the defensin family named *Cg*-Defm, was identified from the oyster mantle [4]. Then, two additional defensins named *Cg*-Defhs (*Cg*-Defh1 and *Cg*-Defh2)have been characterized from hemocytes [5]. Both *Cg-defm *and *Cg-defhs *appear to be continuously expressed in different tissues. More recently, a new peptide, *Cg*-Prp, which belongs to the proline-rich AMP family, has been found in hemocytes [6]. Whereas *Cg*-Prp displays alone a weak *in vitro *antimicrobial activity, it revealed synergistic activity with *Cg*-Defm against both Gram positive and negative bacteria [6]. Additionally, a member of the bactericidal/permeability-increasing protein (BPI) family has been characterized [7]. *Cg-bpi *expression is induced in hemocytes after oyster bacterial challenge and constitutive in various tissue epithelia of unchallenged oysters. Similar to vertebrate BPIs, the oyster BPI binds LPS, displays bactericidal activity against Gram negative bacteria and increases the permeability of the bacterial cytoplasmic membrane [7].

High levels of sequence diversity were reported to be a characteristic of several antimicrobial effectors pertaining to the innate immunity system of invertebrates [8]. The functional significance of this diversity has been addressed in some cases. Multiple variants of lectins from the horseshoe crab *Carcinoscorpius rotundicauda *are able to recognize and differentiate bacteria and fungi [9]. The AMP drosomycin from *Drosophila melanogaster *presents six isoforms with different antifungal activities [10]. Thus, diversification of antimicrobial effectors by accumulation of multiple variations around an originally unique form may provide significant means of acquiring microbial target specificity [11], concerned in the evolutionary arms race between pathogens and their hosts.

In this work, we have investigated the diversity of three oyster antimicrobial effectors and shown the existence of a high diversity in the transcript and genomic sequences of three antimicrobials, two peptides (*Cg-prp *and *Cg-defs*) and one protein (*Cg-bpi*). We also evidence distinct phylogenies between them and propose the existence of a combination of different genetic mechanisms from which sequence variability arises.

## Results

### C. gigas antimicrobial peptides and BPI protein show distinct phylogeny conformations

**Figure 1:**
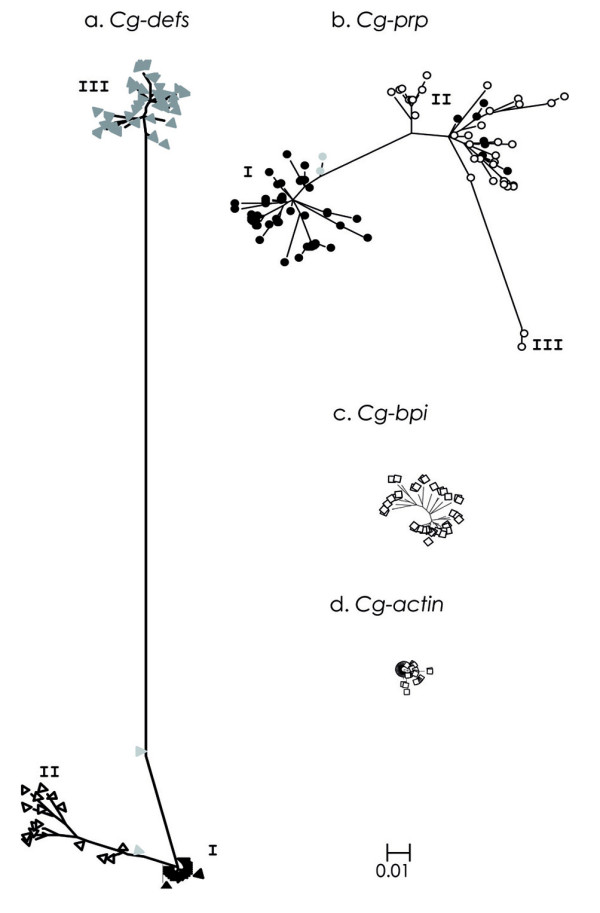
**Phylogenies from CDS of three antimicrobial effectors and non immune gene from *C. gigas***. **a**. *Cg-defs*: I. *Cg-defh1 *(black triangles); II. *Cg-defh2 *(white triangles) and III. *Cg-defm *(dark gray triangles). Recombinant sequences between *Cg-defh1 *and *Cg-defh2 *are indicated (light gray triangles). **b**. *Cg-prp*: I. *Cg-prp *short forms (black circles), II. *Cg-prp *short and long forms, and III. *Cg-prp *long forms (white circles). *Cg-prp *recombinant sequences are indicated (gray circles). **c**. *Cg-bpi *(white rhombus) and **d**. *Cg-actin *(white squares). Construction of cladograms were performed with PHYML v2.4.4 using the models GTR+Γ for *Cg-defs*, HKY85+Γ for *Cg-prp*, TN93+I for *Cg-bpi *and F84+Γ for *Cg-actin *determined by the Akaike information criterion. For accurate comparison between the four genes, cladograms were drawn in the same scale.

To characterize the diversity of all the genes, i.e. the two antimicrobial peptides *Cg-defs *and *Cg-prp*, the antimicrobial protein *Cg-bpi *and the non immune gene *Cg-actin*, we performed an exhaustive sequencing of cloned PCR products and collected sequences in the Sigenae database http://www.sigenae.org/. Finally, we got a total of 256 cDNA sequences and 60 gDNA sequences that we aligned and used to build phylogenies (Table 1). Alignments are available as Popset in GenBank; cDNA sequences of *Cg-prp*, *Cg-defs*, *Cg-bpi *and *Cg-actin*: FJ669353-FJ669402, FJ669323-FJ669352, FJ669296-FJ669322, FJ669287-FJ669295 respectively and gDNA sequences of *Cg-prp *and *Cg-defs*: FJ669252-FJ669286 and FJ669403-FJ669423 respectively. We obtained for both AMPs, *Cg-prp *and *Cg-defs*, clearly structured phylogenies where sequences clustered in distinct groups, whereas *Cg-bpi *and *Cg-actin *sequences formed star-like phylogenies instead (Figure 1). On this basis, we identified three distinct and more or less distant groups of *Cg-defs *(Figure 1a, group I, II and III), each one composed by multiple variants of *Cg-defh1*, *Cg-defh2 *and *Cg-defm*, respectively.

**Table 1:** Number of nucleotide sequences and general polymorphism values of antimicrobial effectors and non immune gene

	*Cg-prp*	*Cg-defm*	*Cg-defhs*	*Cg-bpi*	*Cg-actin*
PCR gDNA	35	11	14	0	0
PCR cDNA	57	40	43	36	40
*in silico*	12	2	15	1	10
total	104	53	72	37	50
different*	50	18	12	28	9

Length (bp)	177/183	195/219	195	1431	1128
SNP ratio 3	4.06	12.9	5.2	16.8	66.3
Hd	0.983	0.857	0.454	0.987	0.093
Pi	0.044	0.009	0.022	0.009	0.002

*N° of sequences with a minimum of 1 nucleotide difference founded at least two times (PCR and/or *in silico *searches)

SNP ratio: ratio of the number of polymorphic sites to the length of CDS i.e. reflect the density of polymorphic sites

Hd: Proportion of different sequences

Pi: nucleotide diversity

Furthermore, we uncovered two lengths of transcripts for *Cg-defm*, one corresponding to the coding sequence (CDS) of 195 bp previously described [4] and a longer one identified here for the first time with a CDS of 219 bp. Similarly, we identified two lengths of transcripts for *Cg-prp*, the one already described with a CDS of 183 bp [6] and a novel shorter one with an indel of 6 nucleotides in the C-terminal region. Likewise to *Cg-defs*, *Cg-prp *sequences are distributed into three different groups (Figure 1b). Group I is only composed of short sequences, group II of long and short sequences, while group III contained only two long and highly divergent sequences. In addition, whenever we got genomic sequences, they are evenly distributed in every group. In contrast to both AMPs, only one group of sequences and homogeneous sequence lengths were obtained for *Cg-bpi *(Figure 1c) and *Cg-actin *(Figure 1d).

The four genes were compared considering the total nucleotide diversity (Pi) from all identified sequences for each gene, their haplotype diversity (Hd) and the mean number of Single Nucleotide Polymorphism (SNPs) in the coding region. Comparison between these values gave evidence of different constraint forces acting on each gene (Table 1). Besides, the average density of SNPs identified for *Cg-actin *was in agreement with SNP values estimated for the genome of *C. gigas *of one SNP every 60 bp, also characterised by direct sequencing of PCR products of several genes [12].

Additionally, the comparison of mean nucleotide diversity (Pi) and the mean nucleotide divergence between groups of each AMP showed that *Cg-prp *presents more diverse but less divergent groups compared to *Cg-defs*. *Cg-prp *presents Pi values of 0.023, 0.035 and 0.006 within group I, II and III, respectively, whereas *Cg-defs *groups present lower Pi values of 0.001, 0.02 and 0.009 within group I, II and III, respectively. Interestingly, *Cg-defh1 *(group I) appears to be highly conserved as compared to *Cg-defh2 *(group II). *Cg-bpi *and *Cg-actin*, which both yield an unstructured phylogeny, present a Pi of 0.009 and 0.002 respectively.

Besides, values of mean nucleotide divergences between groups of AMPs show a higher divergence of *Cg-defs *groups compared to *Cg-prp *ones. *Cg-defs *present mean divergence values of 0.058 between group I and II; 0.214 between group I and III and 0.223 between group II and III, while *Cg-prp *present lower values of 0.063 between group I and II; 0.101 between group I and III and 0.081 between group II and III. This phylogenetic analysis indicates that *Cg-defm *is definitively distinct from *Cg-defhs*. We then separate *Cg-defm *and *Cg-defhs *for further polymorphism analysis.

### Antimicrobial peptides are encoded by multigene families with different gene structures

The evidence of different groups revealed by AMP phylogenies and the number of genomic sequence variants found in one single individual strongly suggest the existence of multiple gene copies. We have therefore estimated the gene copy number of the four molecules analysed in this study. The gene copy number estimation on 3 individuals revealed that C*g-prp *and *Cg-defs *are both represented by a high number of copies with mean values of 13 and 48 copies, respectively. It must be noticed that the primers used for this quantification did not allow the distinction between the different defensins. Thus, this value represents the sum of *Cg-defhs *and *Cg-defm *copies. In contrast, *Cg-bpi *is found as a single copy gene. *Cg-actin *is represented by a higher copy number (mean value of 168 copies), and as for *Cg-defs *and *Cg-prp*, its number of copies varies among the three oysters analysed (Table 2).

**Table 2:** Estimation of gene copy number of antimicrobial effectors and non immune gene by real time PCR

gene	Sample	Primer efficiency (E)		Calculated copy number		Estimated copy number
		**Mean**^**1**^	**SD**	**Mean**^**2**^	**SD**	

***Cg-bpi***	pDNA	2.01	± 0.006			
	gDNA	2.06	± 0.060	0.95	± 0.10	**1**

***Cg-prp***	pDNA	1.99	± 0.008			
	gDNA	2.03	± 0.040	12.59	± 0.99	**11 - 13**

***Cg-defs***	pDNA	2.02	± 0.004			
	gDNA	2.03	± 0.010	47.46	± 6.4 3	**41 - 53**

***Cg-actin***	pDNA	1.99	± 0.030			
	gDNA	2.07	± 0.040	168.04	± 10. 4	**156 - 176**

gDNA: genomic DNA from *C. gigas*

pDNA: plasmid DNA used as standard

^1 ^Mean of primer efficiency (E = 10^-slope^) from three technical replicates

^2 ^Mean of three biological replicates

SD: standard deviation

**Figure 2:**
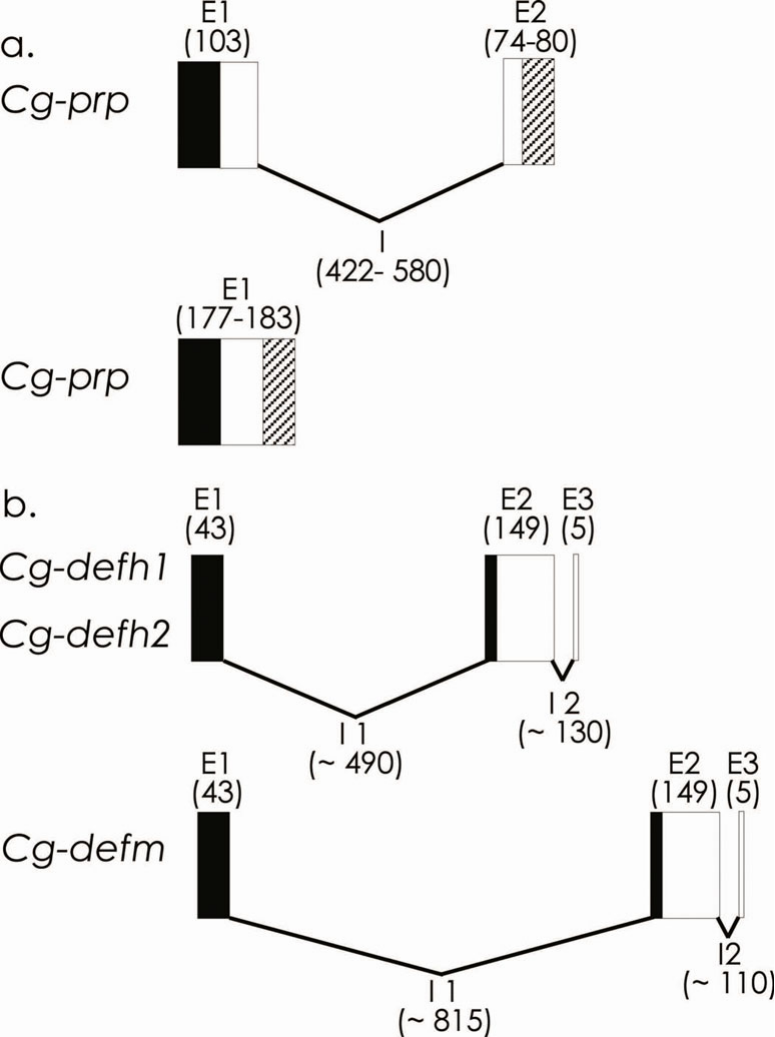
**Gene organization of *Cg-defs *and *Cg-prp *from *C. gigas***. **a**. *Cg-prp *genomic structures. Black boxes indicate signal peptide region; white boxes indicate anionic domain region and striped boxes indicate cationic domain region. **b**. *Cg-defhs *and *Cg-defm *gene structures. Black boxes indicate signal peptide region and white boxes indicate mature peptide region. Numbers indicate the length of exons and introns (bases pairs). Note the gene size is not to scale.

Because both *Cg-defs and Cg-prp *AMPs are present as multigene families, we also characterized their gene structure by exhaustive gDNA sequencing from one individual. The genomic organization of *Cg-prp *and *Cg-defs *revealed two different structures for *Cg-prp *and one single structure for *Cg-defs *(Figure 2). The two structures of *Cg-prp *correspond either to genes with or without an intron, both arrangements coding for the long and short peptide variants. All *Cg-prp *genes containing the intron have the same exonic organization. Exon 1 contains the sequence encoding the signal peptide and half of the residues of the anionic domain (103 bp) while exon 2 encodes the end of the anionic domain and the cationic domain region (177-183 bp) (figure 2a). Nevertheless, we identified several indel events between introns of long and short variants. In addition, intron sequences of short *Cg-prp *variants showed length variations ranging from 571 to 588 bp and multiple SNPs. In contrast to *Cg-prp*, we found a single genomic structure for *Cg-defs*. Exon 1 contains the sequence encoding part of the signal peptide (43 bp) and exon 2 encodes the remaining part of the signal peptide region and the mature peptide region (149 bp), except for the last two residues encoded by exon 3 (5 bp) (figure 2b). However, no homology was found between intron sequences of *Cgdefm *and *Cg-defhs*, confirming thus the very ancient divergence time between the two lineages of these genes. Moreover, introns between *Cg-defm *and *Cg-defhs *differ by length variations. *Cg-defs *and *Cg-prp *AMP genes contain canonical GT/AG splicing recognition sequence located at the end of each intron.

### Antimicrobial peptides and protein variants are produced by several mechanisms of diversification

**Figure 3:**
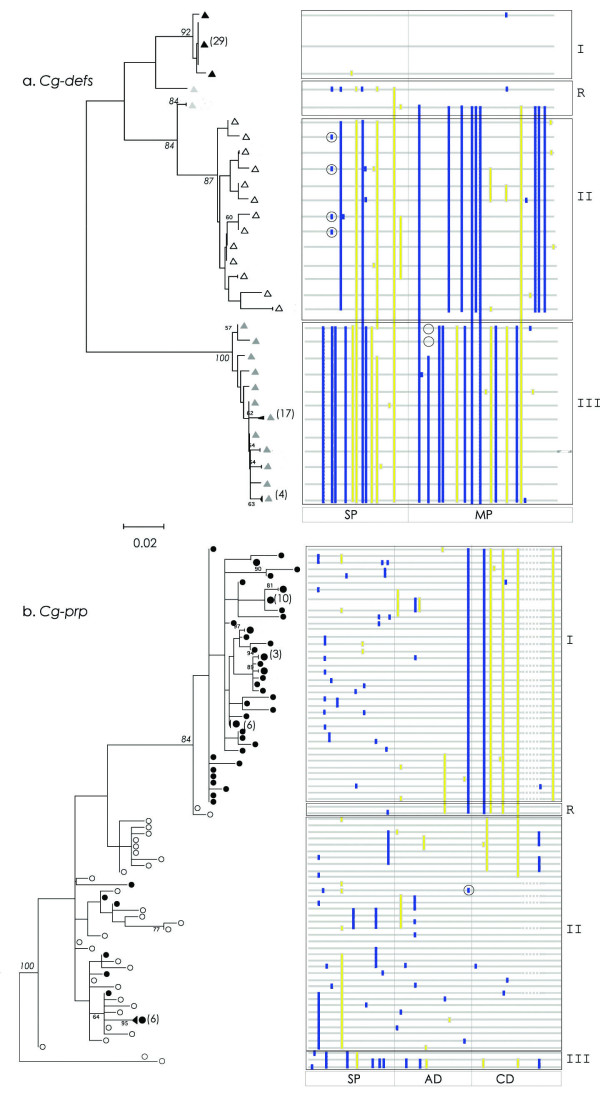
**Cladograms *Cg-defs *and *Cg-prp *and schematic representation of aligned CDS with synonymous and nonsynonymous substitutions**. **a**. *Cg-defs*. **b**. *Cg-prp*. Identical sequences are grouped and the number is indicated at the right of each branch of cladograms and sequence symbols are the same as in figure 1. Nonsynonymous (blue boxes) and synonymous substitutions (yellow boxes) are localized over schematic alignment. Different groups of sequences (I, II and III) and obvious recombinants (R) are boxed. Indel events are represented by dark lines (*Cg-prp*) and striped box (*Cg-defs*) and circles indicate parallel homoplasic mutations. SP: signal peptide; MP: Mature peptide; AD: Anionic Domain; CD: Cationic Domain. For accurate comparison between AMPs, cladograms were drawn in the same scale.

The cDNA and gDNA sequence diversity and the variable gene copy number observed between *C. gigas *families of antimicrobials prompted us to investigate the genetic processes that participated to the diversification. Thus, we analyzed the repartition of synonymous and non synonymous substitutions in each group from AMP phylogenies (figure 3). Each group of *Cg-defs *presents a specific distribution of substitutions all along the CDS (figure 3a). Thus, *Cg-defh1 *is the most overall conserved antimicrobial peptide, with only a few numbers of substitutions. *Cg-defh2*, in contrast, presents a high number of synonymous substitutions and *Cg-defm *presents a profile which differs from hemocyte defensins with higher number of nonsynonymous substitutions. *Cg-prp *shows a different profile compared to *Cg-defs *in terms of the distribution of substitutions. Indeed, the substitutions localized in the signal and anionic peptide region are shared by groups, while substitutions in the cationic peptide region are specific of each group (figure 3b). Altogether, these results show the existence of different constraint levels between and within the groups of AMPs (*Cg-defs and Cg-prp*).

Sequence alignments gave also evidence of several genetic mechanisms of diversification such as recombination and parallel mutations leading to phylogenetic homoplasy. Clues of recombination were detected by the visual inspection of alignments and by the construction of cladograms using different domains of each antimicrobial (data not shown) that lead to inconsistent clustering of sequences: several sequences of *Cg-defhs, Cg-prp *and *Cg-bpi *moving from one clade to another according to the domain used, suggesting past recombination events between members of different groups. We then calculated the minimum number of recombination events (Rm) for all genes. *Cg-defhs*, *Cg-prp *and *Cg-bpi *showed Rm values of 5, 6 and 10, respectively. In contrast, we did not find any evidence of recombination for *Cg-defm *and *Cg-actin*. Because of the large difference in size between our genes and since the probability of recombination is proportional to the length of each sequence, we calculated a ratio of Rm/CDS length for a correct comparison between AMPs and *Cg-bpi *protein. The ratios of Rm/CDS length for *Cg-defhs*, *Cg-prp *and *Cg-bpi *were 0.030, 0.027 and 7 × 10^-3 ^respectively, which gave evidence of a higher probability in AMPs to present recombination between alleles compared to *Cg-bpi*.

In addition to the increase in the gene copy number described above, other processes that participate in AMP diversification are indel events which produce length variations in the CDS. Long variants of *Cg-defm *are obtained by an indel of two nucleotides at the 3'end (position 191) which changes the open reading frame and generates a longer molecule of 24 nucleotides, coding for a peptide of 73 amino acids with eight extra amino acids compared to the original form. For *Cg-prp*, the two lengths are generated by an indel of six nucleotides near the 3'end (position 171), corresponding to a repetition of the precedent nucleotides. These forms code for peptides of 59 and 62 amino acids.

### Antimicrobial peptides and protein show a great number of variants and AMP diversity is shaped by directional selection pressure

**Figure 4:**
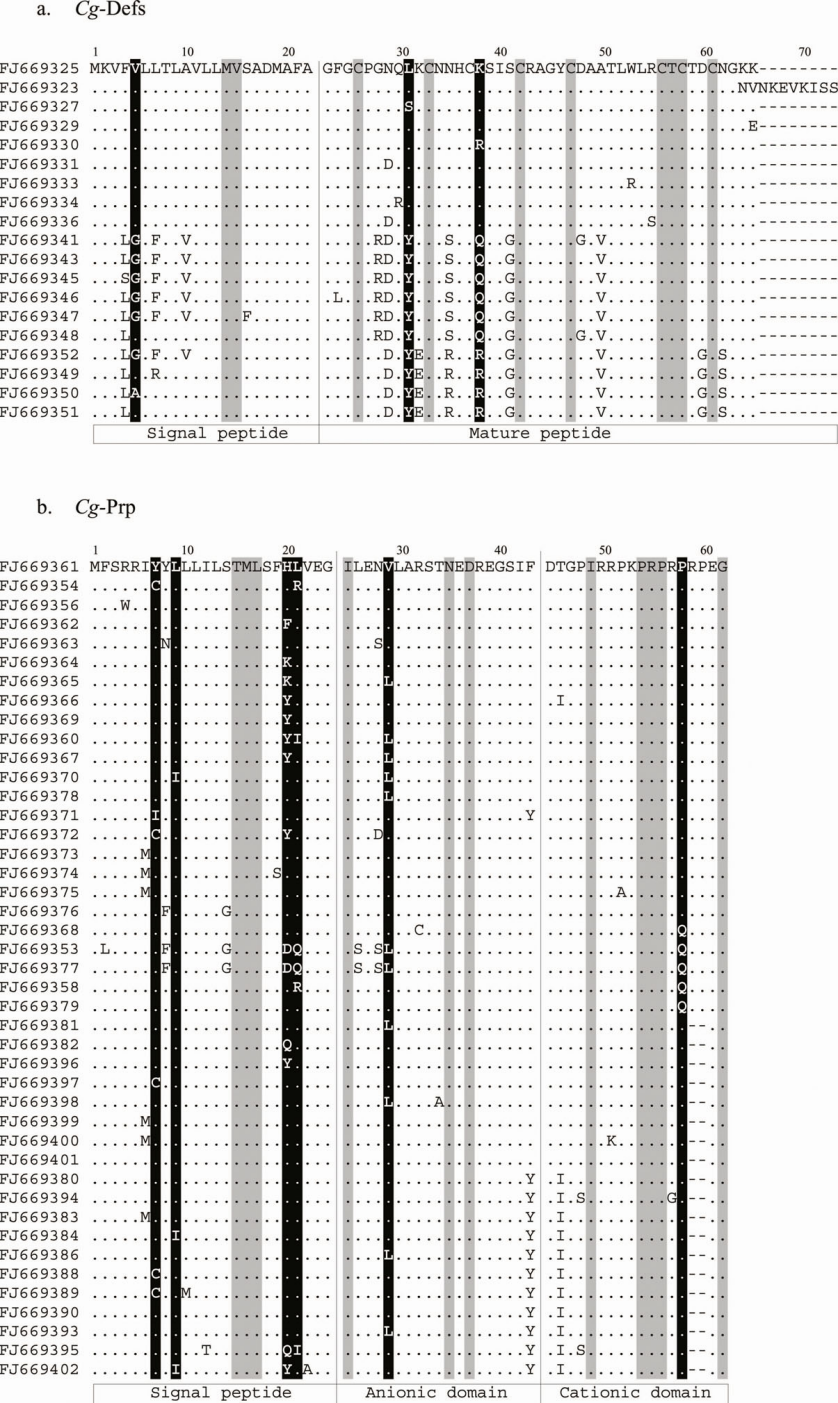
**Alignments of amino acids sequences of putative isoforms for *Cg-defs *and *Cg-prp *from *C. gigas***. **a**. *Cg*-Defm (FJ669323-FJ669336) and *Cg*-defhs (FJ669341- FJ669352). **b**. *Cg*-Prp (FJ669354- FJ669402). Highlighted sites showed evidence of positive (in black) or negative (in gray) selection (*p *< 0.05). Dots indicate identical residues and gaps (-) were introduced to obtain maximum sequence identity. The different domains are indicated in white boxes at the bottom of alignments.

From all sequences analyzed in this study, we identified 43 variants of *Cg*-Prp, 24 variants of *Cg*-BPI, 9 variants of *Cg*-Defm, 10 variants of *Cg*-Defhs and only 5 variants of *Cg*-Actin. Thus, we then analyzed if the sequence diversity has been shaped by directional selection pressures. The ratios of non synonymous to synonymous substitutions (dN/dS) for each codon of the four molecules indicate several sites under negative and positive selection for both AMPs (*Cg*-Defs and *Cg*-Prp), but not for *Cg*-BPI and *Cg*-Actin (Figure 4). To obtain dN/dS ratios, we first excluded all obvious recombinant sequences. Then, to assess the significance of the findings, we carried out a likelihood ratio test (LRT) and considered only codons showing significantly negative and positive selection (*p *< 0.05). Ten and 11 residues showing significant negative selection were identified in *Cg*-Defs and *Cg*-Prp respectively. For both AMPs, we detected a lower number of positively selected sites (3 and 6 residues in *Cg*-Defs and *Cg*-Prp respectively).

In *Cg*-Defs, the signal peptide is well conserved for each class of defensin. Besides, the cleavage site for signal peptidase and the position of the cysteines is completely conserved in all variants (Figure 4a). We identified a high number of amino acid substitutions between *Cg*-Defs and several substitutions occurring in the mature peptide which change the physicochemical properties of the encoded amino acid. Thus, the polarity and/or charge of amino acids are modified in sites 28 (Gly/Arg), 29 (Asn/Asp), 31 (Leu/Tyr), 32 (Lys/Glu), 35 (Asn/Ser/Arg) 38 (Lys/Gln/Arg), 41 (Ser/Gly), and 60 (Asp/Gly). In contrast, substitutions occurring in the signal peptide do not modify the physico-chemical properties of the encoded amino acid. Interestingly, long forms of *Cg*-defm present two extra positive charged amino acids in position 67 and 70 (Lys) and one extra negative charged amino acid in position 68 (Glu).

In *Cg*-Prp, the signal peptide is the most variable region and the cleavage site is completely conserved among all variants, but the putative chymotrypsin cleavage site between the anionic and cationic domain presents an amino acid variation in position 43 (Tyr/Iso) (Figure 4b). Although this mutation is found in both long and short variants, the chymotrypsin cleavage site remained unchanged. Amino acid substitutions are identified in both signal and mature peptide region and they mainly concern the polarity of residues, like sites 7 (Tyr/Ile/Cys), 8 (Tyr/Phe), 14 (Ser/Gly), 26 (Leu/Ser), 45 (Ile/Tyr), 47 (Ser/Pro) and 57 (Pro/Gln). We also found complex sites with four and six different amino acid substitutions per site in which polarity and charge are modified, like sites 20 (His/Phe/Lys/Tyr/Asp/Gln) and 21 (Leu/Arg/Ile/Gln).

## Discussion

Our results showed the existence of high and variable sequence diversity of *C. gigas *antimicrobial peptides (two AMPs,*Cg-prp *and *Cg-defs*) and protein (*Cg-bpi*), produced by a combination of different genetic mechanisms. Phylogenetic trees constructed with CDS from transcripts and genomic sequences showed a distinct phylogeny shape for each gene. The antimicrobial protein *Cg-bpi *expressed in hemocytes presents higher number of variants compared to non immune gene *Cg-actin*, but it is less diverse than AMPs. In contrast to both AMPs, *Cg-bpi *variants fall into one single group encoded by one single gene copy, being in accordance with the one copy present in human [13]. Thus, the sequence diversity observed for *Cg-bpi *results from allelic polymorphism only, with occasional recombination between alleles at a lower frequency when compared to AMPs. Though sequences obtained by PCR approach come from relatively limited sample size (N = 144) but with a large spectrum of oyster origin and challenge conditions, the sequence sampling saturation curve (plot of the number of different haplotypes to the total number of sequences performed) have a sigmoid shape indicating that sequencing effort was sufficient in our population to approach the plateau of saturation for each gene (data not shown). Furthermore, we have complemented the sample size by adding all the *C. gigas *ESTs available in databases. These ESTs come from more than 20 cDNA libraries (different tissues, experimental conditions and developmental stages) retrieved in the last decade. The combination of these two distinct sources of sequences is probably not exhaustive but covers a large enough range of variants to give a good insight of the overall polymorphism of the genes.

*Cg-prp*, which is expressed only in hemocytes, presents the highest number of transcript variants that are distributed into three groups. This partitioned phylogeny results in part from group specific mutations affecting only the cationic region. These key mutations in the potential active domain have segregated into two major lineages that are further diversified on other sites. There, high polymorphism in signal peptide and anionic region induces high sequence diversity within each group. Interestingly, we observed two very divergent sequences forming a third distinct group. Such a long branch in the phylogeny leading to few sequences may be indicative of an ongoing or recent process of neo-functionalization or subfunctionalization. These kinds of phenomena are often invoked in the course of evolution of duplicate genes [14], here supported by the finding of multiple copies for *Cg-prp*. In addition to this, sequence polymorphism of *Cg-prp *is produced by recombination and convergent mutations leading to phylogenetic homoplasy. Besides, *Cg-prp *is encoded by a lower number of copies than *Cg-defs *but shows higher number of different transcripts, indicating that allelic polymorphism associated with higher recombination frequency is important for this antimicrobial effector. Noticeably, intronless genes were found for *Cg-Prp *as previously described in other organisms where it was demonstrated to accelerate mRNA maturation process [15]. On the opposite, intron presence was also described to have positive effect on transcript stability [16]. Nevertheless, the role of different genomic structures and its correlation with the antimicrobial function remains unclear.

*Cg-defs *expressed in hemocytes and mantle tissue exhibit the highest divergence between groups but a lower diversity within each one. Moreover, each *Cg-defs *group displays different conservation level, showing that *Cg-defh1 *is less diversified than *Cg-defh2*, *Cg-defm *and even *Cg-actin*. Since we collected the different *Cg-defs *in the same conditions, we can rule out a technical bias inducing a highly redundant sequencing of the same molecule *Cg-defh1 *and leading to a skewed appreciation of the relative variability of each group. Thus, two major hypotheses could explain this extremely low level of variability: (i) *Cg-defh1 *appeared recently and is not yet diversified albeit frequent in the genome we explored, or (ii) *Cg-defh1 *could be an older variant but have undergone strong selective pressure. There is no apparent reason why *Cg-defh1 *could be more under selective constraints than the other *Cg-def*s. So the polymorphism reduction it displays is more likely due to a recent selective sweep than to purifying selection acting in a long term manner. We can postulate that this selective sweep could be consecutive to infections by pathogens that *Cg-defh1 *was particularly effective to combat. In addition, the fact that *Cg-actin *also appears more polymorphic at nucleotide level than *Cg-defh1 *is also in favour of a selective sweep on *Cg-defh1*, since it indicates that the polymorphism reduction concerns both the synonymous and the non-synonymous sites while purifying selection would affect mainly non-synonymous mutations. However, further studies are needed to clarify this point.

*Cg-defs *also present recombination parallel homoplasic events and a multigene family as observed for *Cg-prp*. Nevertheless, the absence of recombination between *Cg-defm *and *Cgdefhs*, together with the lack of homology between their introns, indicates that these two lineages are separated for a longer time than *Cg-prp*. This could be associated with the tissue specific expression of *Cg-defm *[4] and *Cg-defhs *[5] probably acquired after a duplication event followed by sub-functionalization. The tissue specific expression has already been described for several AMPs like mice α-defensins, which might be linked to differences in innate immune functions [17]. Likewise, sequence diversification of AMPs by gene duplication has been reported for vertebrates and invertebrates [18,19].

It is remarkable that gene duplication, as well as strong selective pressure (either positive or negative), did not affect identically the genes that we have studied; the antimicrobial peptides,*Cg-defs *and *Cg-prp*, have been a target for both, while antimicrobial protein *Cg-bpi *was not. This may reflect a difference in the way these two types of molecules participate in the immune response.

Concerning the sites under selection, the sites subject to purification found for both AMPs correspond to residues under strong functional constraints. In *Cg*-Defs, seven of eight Cys residues are under negative selection and Cys residues are known to be essential for the antimicrobial function of these peptides [20]. In *Cg*-Prp, the conserved Pro-Arg motif has been involved in antimicrobial activity of this class of AMPs [21] and the conserved C-terminal Gly is expected to be eliminated, obtaining a C-terminally amidated peptide which would increase its cationic properties [6].

In addition to these constrained sites, sites subject to diversifying selection of the AMPs could also have a role in antimicrobial function. In *Cg*-Defs, positively selected sites concern the modification of the charge and/or polarity of certain amino acids, and these changes could probably affect the affinity of the peptides to bacterial membranes. This finding is in accordance with several sites in human beta-defensin 1, in which amino acid charge is directly involved in the antimicrobial activity [22]. In *Cg*-Prp, from all positively selected sites, we identified a site in the active domain where a change of a Pro toward a Gln occurred. Because the activity of proline-rich AMPs [23] relies of course on proline residues, the functional characterization of these variants in terms of efficiency could help in understanding their role in the antimicrobial response. The functional significance of other positively and negatively sites along the coding region of *Cg-defs *and *Cg-prp *remains unknown and needs further investigation. Nevertheless, we propose, following several authors [24,25] that strong selective pressures on AMPs are directed toward the acquisition of novel microbial target specificities.

Evidence for directional selection driving evolution has been addressed in several cases [26-28] and it is consistent with the general hypothesis of co-evolution or "arms race" [29]. In this hypothesis, the pathogen evolves continuously to escape from the immune response of host and, consequently, the immune system of host evolves to improve barriers against pathogens. Thus, such interactions might provide rapid evolution in genes concerned in host-pathogen interactions. The emergence of *Cg-defh1*, a form of *Cg-defh*s that seems to have largely spread out in the oysters we analyzed, could be a good illustration of this.

Besides the sites under directional pressures, both AMPs show different lengths of mature peptide which could modify their antimicrobial activities. In one hand, *Cg*-Defm long variants present a longer C-terminal tail with eight extra amino acid residues that could influence the affinity to the bacterial membranes. Indeed, different C-terminal tails of alpha-defensins from rat are already described and are predicted to be exposed to the surface where they might confer a different antimicrobial spectrum [30]. On the other hand, the two lengths of *Cg*-Prpare produced by an indel of an Arg-Pro motif in the cationic domain, which is described to be involved in the antimicrobial activity of proline rich AMPs [23]. All these differences between antimicrobial effectors could be related to specific antimicrobial functions and result from co-evolution between host and pathogen species.

## Conclusions

All these data support the hypothesis that oysters, continuously exposed to an environment rich in microorganisms, have developed specific strategies of innate immunity to increase the antimicrobial defense upon infection through effector diversification. In addition, considering the synergistic activities already reported between the two AMPs, *Cg*-Prp and *Cg*-Defm, [6] and between several isoforms of the same AMP in other species [31,32], the observed diversity could also improve the antimicrobial response through synergic activities. The next step of our work is now to establish the functional significance of this diversity.

## Methods

### Animals

Hemocytes were sampled from *C. gigas *adult oysters under 24 experimental conditions (total of 144 oysters): oysters of three geographic origins (6 oysters per condition; Atlantic coast-La Tremblade, Normandie-Bay des Veys and Mediterranean Sea-Thau lagoon) were exposed to four kinds of bacterial challenges and hemolymph were sampled and pooled at two post challenge times. The bacterial challenges performed by oyster immersion were (1) alive non virulent *Micrococcus luteus *and *Vibrio tasmaniensis *(2.5 × 10^8 ^bacteria/L for each strain), (2) alive virulent *V. splendidus *(5 × 10^8 ^bacteria/L), (3) mix of heat killed virulent *V. splendidus *and *V. aestuarianus *(2.5 × 10^8 ^bacteria/L for each strain) and (4) unchallenged oysters. For each condition, hemolymph was collected at 12 and 24 hours after challenge from the pericardial cavity through the adductor muscle. After hemolymph collection, hemocytes were isolated by centrifugation to discard plasma (700 *g *for 10 min. at 4°C) for further RNA extraction. For RNA extraction of mantle tissue, oysters from the Mediterranean Sea (Thau lagoon) were harvested by dissection. All experimental infections were performed according to the Ifremer animal care guideline and policy.

For the genomic DNA extractions (gDNA), hemocytes were recovered individually as described above from three oysters used for gene quantification and from one oyster used for gene structure determination originating from the Mediterranean Sea (Thau lagoon).

### RNA extraction and cDNA synthesis

Total RNA was extracted from *C. gigas *hemocytes and mantle tissues using Trizol reagent according to manufacturer instructions (Invitrogen™). RNA was then treated with DNAse I (Invitrogen™), 15 min at room temperature and inactivated by heat, 10 min at 65°C. A second precipitation was performed with sodium acetate 0.3 M to eliminated DNAse and degraded DNA. Then, quantification and quality of total RNA were determined using a *NanoDrop *spectrophotometer (*NanoDrop *Technologies) and agarose gel electrophoresis, respectively. Following heat denaturation of 1 μg of total RNA (65°C for 5 min), first strand synthesis was carried out using 50 ng oligo-(dT)_12-18_, 1 mM dNTPs, 1 unit RnaseOUT (Invitrogen™) and 200 units M-MLV reverse transcriptase in reverse transcriptase buffer (Invitrogen™) following the manufacturer protocol.

### Genomic DNA extraction

Genomic DNA (gDNA) was isolated from oyster hemocytes individually, by incubation in buffer (100 mM NaCl, 10 mM Tris-HCl pH 8, 25 mM EDTA pH 8, 0.5% SDS and 0.1 mg/ml proteinase K), 4 h at 50°C, followed by phenol/chloroform extraction. gDNA was precipitated by the addition of cold ethanol (-80°C) and treated with 50 μg/ml RNAse (Invitrogen™), 30 min at 37°C. This was followed by a second DNA precipitation and DNA purity and concentration were determined as described above for RNA samples.

### 5' and 3' RACE, molecular cloning and sequencing

Rapid amplification of cDNA ends (RACE) were performed from hemocytes and mantle tissue RNA to obtain both 5'UTR and 3'UTR of *Cg-defh1 *and *Cg-defh2 *and the 5'UTR of *Cg-defm*. We designed three specific antisense primers from *Cg-defh *cDNA partial coding sequences (CDS) [GenBank DQ400101 and GenBank DQ400102] for the 5' RACE, one specific sense primer for the 3' RACE and three antisense primers for the 5' RACE from *Cg-defm *cDNA CDS [GenBank AM050547]. Primers are listed in table 1. The procedure was performed according to the manufacturer 5'/3' RACE protocol (Roche, Mannheim, Germany). Briefly, 5' RACE for *Cg-defhs *was carried out using 1 μg of total RNA, 1.6 μM of *Cg-defh5' sp1 *antisense primer, 1 mM dNTPs, 1 unit/μl RnaseOUT (Invitrogen™) and 200 units/μl M-MLV reverse transcriptase in reverse transcriptase buffer (Invitrogen™). The cDNA was then treated with two units of RNaseH for 20 min at 37°C. ssDNA product was purified with High Pure PCR Product purification kit (Roche), and a poly A end was added with 1 μL of terminal transferase (80 U/μL) (Promega). The 5'UTR for *Cg-defhs *was obtained after two PCR reactions, the first reaction with the antisense primer *Cg-defh*5'*sp2 *and the oligo (dT) anchor primer (Roche) and the second one with the antisense primer *Cg-defh*5'*sp3 *and the race (dT) anchor primer (Roche). 5'RACE for *Cg-defm *was performed with the three antisense primers as described above. 3' RACE for *Cg-defhs *was performed in the same conditions as 5' RACE but using the oligo (dT) anchor primer (Roche) for first strand synthesis. Only one round of PCR was performed using the specific sense primer *Cg-defh*3 *'sp1 *and race (dT) anchor primer (Roche) to obtain 3'UTR. Each RACE-PCR products was gel purified using a QIAQuick^® ^gel extraction kit (Qiagen, Germany) and cloned using TOPO TA (Invitrogen™) with the pCR 2.1 TOPO^® ^vector. Plasmids were transformed into TOP10 *E. coli *cells, and plasmids were purified using Wizard™ SV miniprep DNA purification kit (Promega). Plasmid sequencing was performed using Big Dye™ Terminator sequencing kit on a DNA sequencer model ABI Prism 3130XL (Applied Biosystems).

### PCR amplification and sequencing from cDNA and gDNA

PCR amplifications from cDNA were performed using specific primers designed in the 5'UTR and in 3'UTR from sequences of *Cg-prp *[GenBank BQ426670], *Cg-bpi *[GenBank AY165040], *Cg-defm *[GenBank AM050547] and *Cg-actin *[GenBank EW779066]. Specific primers for *Cg-defhs *and sense primer for *Cg-defm *were designed in the 5'UTR and 3'UTR from sequences retrieved through RACE assays described above. The sequences of each specific pair of primers are listed in Table 3. *Cg-prp, Cg-defhs, Cg-bpi *and *Cg-actin *were amplified from cDNA synthesised from a pool sample of hemocyte RNAs and *Cg-defm *was amplified from cDNA synthesised from a pool sample of mantle tissue RNAs. PCR reactions were carried out with GOTaq polymerase according to manufacturer instructions (Promega), using 1 μl of synthesized cDNA under the following conditions: 10 min at 95°C, then 35 cycles at 95°C for 30 s, 57°C for 1 min, 72°C for 2 min and a final elongation step at 72°C for 7 min. PCR products were cloned and sequenced as described above.

**Table 3:** List of primers

	Sense primers (5'3')		Antisense primers (5'3')	
**gene**		**PCR amplification from cDNA and gDNA**		

*Cg-prp*	*Cg-prp2F*	CACCATGTTCTCTCGGAGGA	*Cg-prp3R*	TCTCTTCATCAAAACAAAGTCG
*Cg-defm*	*Cg-defm5F*	CCACTTTCTGGTTTGCTGAG	*Cg-defU4R*	TCTTGGTCAGATTCAGWCTGG
*Cg-defh*	*Cg-defh4F*	CTACCAGTTGTTCATACAGAG	*Cg-defU4R*	TCTTGGTCAGATTCAGWCTGG
*Cg-bpi*	*Cg-bpi1F*	CTACCAGTTGTTCATACAGAG	*Cg-bpi1R*	GGATTTAATATATCCGTCTTCTG
*Cg-actin*	*Cg-actin1F*	CTTCACAATGGGAGATGAAGA	*Cg-actin1R*	GTAAACTCCTATCACAGCCAC

		**gene copy number estimation**		

*Cg-prp*	*Cg-prpUq1F*	CCACCATGTTCTCTCGGAGG	*Cg-prpUq1R*	CTTGTAGAACGGGCTAGCAC
*Cg-defs*	*Cg-defUq1F*	CTGCAGACATGGCTTTTGCTG	*Cg-defUq1R*	GTACATCTTGACCAGAGCGTG
*Cg-bpi*	*Cg-bpiq2F*	AGATAGAAATAGGAATGGACGG	*Cg-bpiq2R*	GTTATAGATCCACGCTGCTCC
*Cg-actin*	*Cg-actq2F*	CAGCTATGTAGGAGACGAG	*Cg-actq3R*	CACGGAGTTCATTGTAGAAGG

		**5' and ' RACE**		

*Cg-defh*	*Cg-defh3'sp1*	GCTGGATTTGGGTGTCCG	*race (dT) anchor*	GACCACGCGTATCGATGTCGAC
*Cg-defh*			*Cg-defh5' sp1*	CACAGTAGCCCGCTCTACA
*Cg-defh*	*oligo (dT) anchor*	GACTCGAGTCGACATCGATTTTTTTTTTTTTTTTV	*Cg-defh5' sp2*	CGGACACCCAAATCCAGC
*Cg-defh*	*race (dT) anchor*	GACCACGCGTATCGATGTCGAC	*Cg-defh5' sp3*	CCATYTCTGCAGAAACCATC
*Cg-defm*			*Cg-defm5' sp1*	CATCTTAACCAGAGCGTGGC
*Cg-defm*	*oligo (dT) anchor*	GACTCGAGTCGACATCGATTTTTTTTTTTTTTTTV	*Cg-defm5' sp2*	GGACTTGCAGTGATTGTTGC
*Cg-defm*	*race (dT) anchor*	GACCACGCGTATCGATGTCGAC	*Cg-defm5' sp3*	CATCAGAAGGACAGCTAGTG

Genomic sequences were obtained by PCR amplification on gDNA from one individual sample with the same specific pair of primers used to amplify cDNA (Table 1), in a total volume of 25 μl. Template (100 ng of gDNA) was amplified under the following conditions: 10 min at 95°C, followed by 35 cycles at 95°C for 1 min, 57°C for 1 min, 72°C for 3 min and a final elongation step at 72°C for 10 min. PCR products were cloned and sequenced as described above.

### In silico approach

Sequences previously identified for *Cg-prp*, *Cg-defm*, *Cg-defh1*, *Cg-defh2*, *Cg-bpi *and *Cg-actin *were used for the search of homologous sequences among the 29,745 unique sequences contained in the Sigenae *C. gigas *EST database http://www.sigenae.org/[33,34].

From all the identified sequences, we kept only sequences with the complete CDS. In addition, to avoid scoring PCR or sequencing artefacts, we have only considered sequences shared between PCR and *in silico *approaches or those observed at least twice in the same approach.

### Gene copy number estimation

For each gene, specific pairs of primers were designed from a conserved region on one exon selected by comparison of all the sequences compiled in this study (Table 3). As standard of gene quantification, we used equimolar amounts of pCR 2.1 TOPO ^® ^vector plasmid DNA (pDNA) containing the inserts of interest which were pooled and diluted in 40 ng of herring sperm DNA. Efficiency of amplification was calculated by serial dilutions ranging from 10^3 ^to 10^9 ^copies per reaction which were tested in duplicate with each primer pair. qPCR assays were then performed in duplicate with four serial dilutions of gDNA (between 5 to 40 ng per reaction) from 3 individuals separately, and we used the concentration of 20 ng to estimated the gene number. qPCR was carried out on the LightCycler 480 System (Roche). The reaction mixture consisted of LightCycler 480 master mix (1×) and 0.5 μM of each primer and was submitted to: 10 min of denaturation at 95°C, 40 cycles of 10s of denaturation at 95°C, 20s of annealing at 60°C and 20s of extension at 72°C, and fluorescence detection. After an initial 10 s denaturation step at 95°C, a melting curve was obtained from a start temperature of 65°C to a final temperature of 95°C, with an increase of 0.06°C/s. The data were analyzed using LightCycler 480 software version 1.5.0.39 and the 2^nd ^derivative max algorithm.

Gene copy number was calculated by absolute quantification. Standard curves for gDNA and pDNA of each gene were constructed from the mean Ct values using linear regression, from which slope and correlation coefficients were calculated. PCR efficiency was determined by plotting the threshold cycle (Ct) as a function of Log10 concentration of the template used. Using an estimation of the *C. gigas *genome size of about 823 Mb, the number of molecules represented in 20 ng of gDNA was then calculated. Quantification was finally achieved by plotting the measured threshold cycle (Ct) on the standard curve obtained with the serial dilutions of pDNA. pDNA and gDNA can be used similarly as reference molecules if their PCR efficiencies and correlation coefficients are identical [35].

### Sequence data analysis

The multiple alignments were generated using the MAFFT alignment program (http://align.bmr.kyushu-u.ac.jp/mafft/online/server/, version 6) [36]. Prediction of signal peptide was performed with the SignalP program (http://www.cbs.dtu.dk/services/SignalP/, version 3.0) [37]. Construction of phylogenetic trees was performed with PHYML v2.4.4 [38] using the models with the best Akaike information criterion (Phymltest function from the APE package) [39].

On the basis of the uncovered sequence phylogenies, we defined clearly distinct groups of sequences that were used in further polymorphism analyses, whereas sequences with ambiguous positions were not considered. Measure of sequence diversity as the mean nucleotide diversity (Pi) (intra-group) and the mean nucleotide divergence (inter-groups) values were calculated using the Maximum Composite Likelihood method included in the Molecular Evolutionary Genetics Analysis program (http://www.megasoftware.net/ version 4.0) [40]. Polymorphism values and minimum numbers of recombination events (Rm) were calculated with DNAsp program (http://www.ub.es/dnasp/ version 4.20.2) [41]. The ratio of the rate of non-synonymous substitutions (dN) to the rate of synonymous substitutions (dS) for each codon was calculated with Selecton web server http://selecton.tau.ac.il/index.html[42], based on M8 evolutionary model which allows for positive selection. Statistical significance of results (*p *< 0.05) was assessed using the likelihood ratio test (LRT) which compares the log likelihood of M8 model to the log likelihood of M8a alternative model, that allows for negative and neutral selection.

## Authors' contributions

JdL, EB and YG participated in the conception and design of the study and in its coordination. PS produced the genetic data and carried out the analyses. ED provided advice and support for the analysis and helped design the sequence phylogenies. PS and JdL wrote the manuscript with contributions from all the other authors. All authors read and approved the final manuscript.
